# Genome-wide methylation patterns predict clinical benefit of immunotherapy in lung cancer

**DOI:** 10.1186/s13148-020-00907-4

**Published:** 2020-08-06

**Authors:** Jeong Yeon Kim, Jung Kyoon Choi, Hyunchul Jung

**Affiliations:** 1 Department of Bio and Brain Engineering, KAIST, Daejeon, 34141 Republic of Korea; 2 Penta Medix Co., Ltd., Seongnam-si, Gyeongi-do, 13449 Republic of Korea; 3 Cancer Ageing and Somatic Mutation Programme, Wellcome Sanger Institute, Cambridge, UK

**Keywords:** Immunotherapy, Lung cancer, Methylation

## Abstract

**Background:**

It is crucial to unravel molecular determinants of responses to immune checkpoint blockade (ICB) therapy because only a small subset of advanced non-small cell lung cancer (NSCLC) patients responds to ICB therapy. Previous studies were concentrated on genomic and transcriptomic markers (e.g., mutation burden and immune gene expression). However, these markers are not sufficient to accurately predict a response to ICB therapy.

**Results:**

Here, we analyzed DNA methylomes of 141 advanced NSCLC samples subjected to ICB therapy (i.e., anti-programmed death-1) from two independent cohorts (60 and 81 patients from our and IDIBELL cohorts). Integrative analysis of patients with matched transcriptome data in our cohort (*n* = 28) at pathway level revealed significant overlaps between promoter hypermethylation and transcriptional repression in nonresponders relative to responders. Fifteen immune-related pathways, including interferon signaling, were identified to be enriched for both hypermethylation and repression. We built a reliable prognostic risk model based on eight genes using LASSO model and successfully validated the model in independent cohorts. Furthermore, we found 30 survival-associated molecular interaction networks, in which two or three hypermethylated genes showed significant mutual exclusion across nonresponders.

**Conclusions:**

Our study demonstrates that methylation patterns can provide insight into molecular determinants underlying the clinical benefit of ICB therapy.

## Introduction

Over the past several years, the number of non-small-cell lung cancer (NSCLC) patients treated with immune checkpoint blockade (ICB) therapy has increased at a fast rate due to its proven efficacy in treating NSCLC [1]. In particular, ICB therapy targeting programmed death-1 (PD-1) and programmed death-ligand 1 (PD-L1) has demonstrated effectiveness in dramatically improving NSCLC patient survival [2]. However, ICB therapy has varying degrees of effect in patients. Previous studies showed that only a small subset of NSCLC (< 20%) patients can benefit from these novel agents [3, 4]. Thus, it is crucial to unravel the molecular determinants of the response to ICB therapy. To select patients who are likely to respond to this therapy, many genomic and transcriptomic biomarkers have been proposed, such as tumor mutational burden (TMB) [5] and the expression of key genes (e.g., *PD-L1*) [6, 7]. However, these biomarkers are not sufficient to accurately predict the response to ICB therapy. Beyond genomic and transcriptomic biomarkers, epigenetic aberrations have been reported to be associated with response to ICB therapy [8].

Aberrant DNA methylation has been recognized as a crucial factor in lung carcinogenesis [9]. In particular, the inhibition of tumor suppressor expression by promoter hypermethylation is a common event in NSCLC [10]. Thus, DNA methylation-based biomarkers have been extensively studied for predicting prognosis and response to conventional therapy [11, 12]. Silenced immune-related genes by promoter hypermethylation have been recently found to be predictive of ICB therapy response [13–15]. Given that the loss of functions in immune-related genes by genomic mutations (e.g., truncating mutations and copy number deletion) account for primary and acquired resistance to ICB therapy [16–18], we hypothesize that the silencing of immune genes through promoter hypermethylation could be one of the key mechanisms resulting in immune evasion. To comprehensively explore DNA methylation aberrations associated with the response to ICB therapy, we systematically analyzed DNA methylation markers predictive of ICB benefit by investigating genome-wide methylation data (Illumina 850K/EPIC platform) from a total of 141 NSCLC patients receiving ICB therapy. We discovered diverse predictive DNA methylation markers by building a lasso regression prediction model and investigating the molecular network of mutually exclusive promoter-hypermethylated genes. As a result, we suggest that DNA methylation is a promising candidate for biomarkers predicting the clinical response of ICB therapy.

## Results

### Differential DNA methylation pattern between nonresponders and responders

To comprehensively assess the genomic characteristics of our ICB therapy cohort (*n* = 60; Supplementary Table 1), we performed genome-wide DNA methylation (850K Infinium Methylation EPIC Array) and exome-seq assays. First, we evaluated the association of patient survival and known predictive genomic biomarkers, such as TMB, neo-antigen load, aneuploidy level, and PD-L1 expression (Supplementary Figure 1). Remarkably, our cohort’s clinical benefit was not predictable with these biomarkers; for example, our cohort did not show a correlation between mutation burden and progression-free survival (PFS). This independent pattern was preserved even after excluding a patient with an outlier TMB. Moreover, a high aneuploidy level was not significantly correlated with shorter PFS. We also examined known mutation signatures in NSCLC with respect to patient’s PFS because APOBEC mutational signatures are reported to be enriched in NSCLC patients with durable clinical benefit [19, 20]. None of the mutation signatures were related with patient survival in our cohort.

**Fig. 1:**
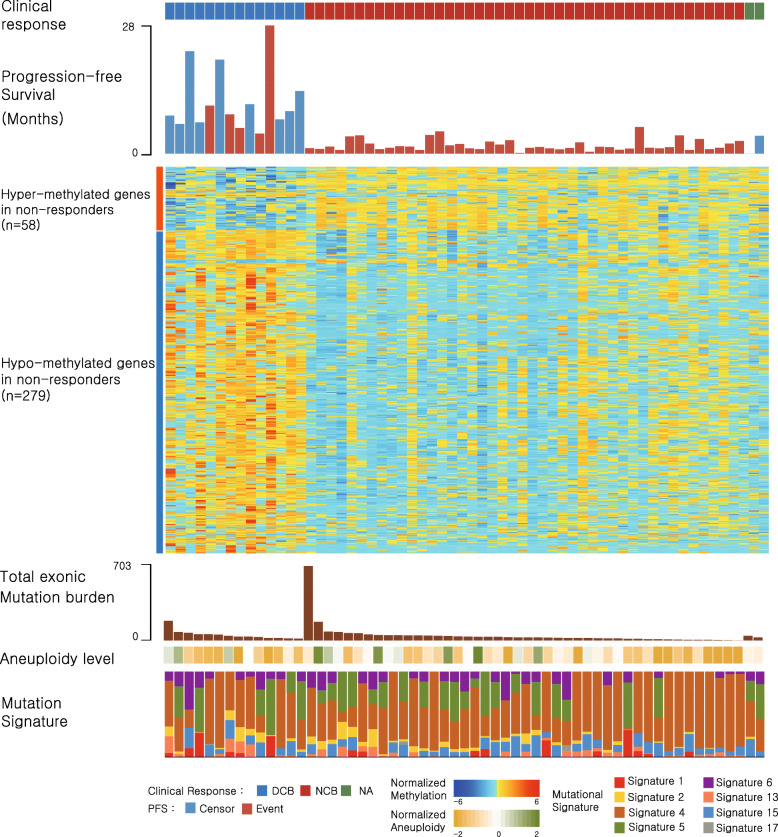
Genomic characteristics of nonresponders and responders. The patients are ordered according to their clinical classification: durable clinical benefit (DCB; responders) on the left, non-durable benefit (NDB; nonresponders) on the middle, and unknown on the right. Within the groups, samples are ordered by decreasing mutation burden. Heatmap (normalized by *z* score transformation per row) shows differentially methylated promoter genes between responders and nonresponders (*P* < 5 × 10^−5^ by *t* test). The total exonic mutation burden, aneuploidy level, and mutation signature obtained from whole-exome sequencing data are shown below the heatmap

Unlike these known biomarkers, the DNA methylation level illustrated its power to distinguish clinical response (Fig. 1). We found 65 and 377 differentially methylated regions (DMRs) and probes, respectively, between responders and nonresponders. A majority of DMRs (97%; *n* = 63) is hypermethylated in nonresponders, and they are concentrated in promoter regions (Supplementary Figure 2), suggesting the potential of a strong local hypermethylation pattern regulating biological functions. On the contrary, the hypermethylated probes (*n* = 64) in nonresponders tend to reside more in gene body regions whereas the hypomethylated probes (*n* = 313) are concentrated in promoter regions. To analyze differential methylation patterns at the gene level, we first assigned the most differently methylated promoter probes to their corresponding genes (see “Methods” section). As a result, we found 337 differentially methylated genes, of which 58 and 279 genes are hyper- and hypomethylated in nonresponders, respectively. Interestingly, the hypermethylated genes in nonresponders were significantly enriched in the positive regulation of interferon-gamma secretion and the positive regulation of apoptotic process, whereas the hypomethylated genes were enriched in transcription regulation and DNA-directed RNA polymerase II activity (Supplementary Table 2).

### Promoter hypermethylation leads to the transcriptional silencing of immune pathways

**Fig. 2:**
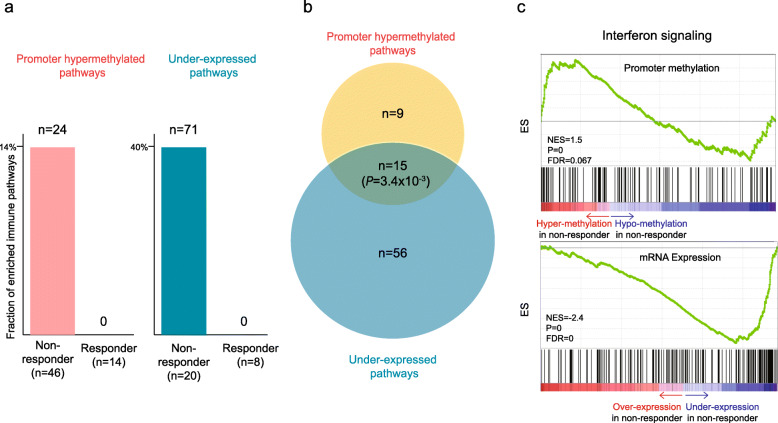
Transcriptional silencing of immune pathways by promoter hypermethylation in nonresponders. **a** Fraction of enriched hypermethylated (left) and under-expressed immune pathways (right) in nonresponders and responders. The number of the pathways showing significant enrichment (FDR < 0.1) by GSEA is indicated above the bars. **b** Relationship between enriched hypermethylated and under-expressed immune pathways in nonresponders. The significance of overlap was determined by Fisher’s exact test. **c** Representative GSEA plots of the overlapped pathways

DNA methylation in promoters is a well-established epigenetic mechanism for the inhibition of gene transcription [21]. To examine the extent to which promoter methylation affects the transcriptional silencing of immune genes, we further analyzed the RNA-seq data on a subset of our cohort (*n* = 28). We then examined promoter hypermethylated and under-expressed pathways between nonresponders and responders using Gene Set Enrichment Analysis (GSEA) [22]. Interestingly, nonresponders had both enriched promoter hypermethylated (*n* = 24, 13.3%; Supplementary Table 3) and under-expressed immune pathways (*n* = 71, 39.2%; Supplementary Table 4), whereas responders did not have any significantly enriched immune pathways, suggesting the down-regulation of immune system in nonresponders (Fig. 2a). We further examined relationship between promoter hypermethylated and under-expressed pathways in nonresponders and found a statistically significant overlap between them (Fig. 2b, *P* = 3.4 × 10^−3^). Representative GSEA plots of the overlapped pathway, namely interferon signaling, are illustrated in Fig. 2c. These results highlight the importance of the transcriptional silencing of immune pathways by promoter-hypermethylation in nonresponders.

### Establishing a methylation-based prognostic prediction model

**Fig. 3:**
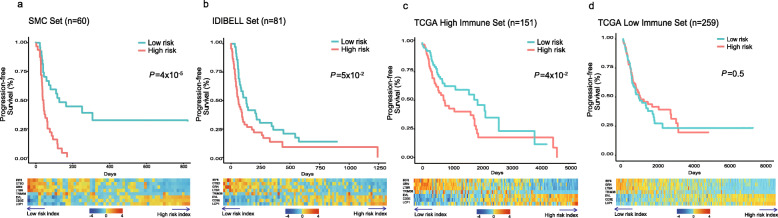
Risk score calculated by Lasso Cox regression models and survival analysis in three different cohorts. **a** Training set (*n* = 60; our cohort). **b** IDIBELL set (*n* = 81). **c**–**d** TCGA high- (*n* = 151) and low-immune pressure cohorts (*n* = 259). Patients in the TCGA cohort were divided into high- and low-TIL cohorts according to mean value of tumor infiltrating lymphocyte (TIL) fraction. Kaplan-Meier survival analyses of the patients are displayed on the top. The patients in each cohort were divided into low- and high-risk groups based on mean of risk index produced by our model (i.e., mean score). *P* values were calculated using the log-rank test. The methylation levels of the eight genes included in our model are shown as a heatmap on the bottom. Methylation values were *z* score normalized per gene. Genes (x-axis) and samples (y-axis) are ordered in increasing order of coefficient and risk score, respectively. Methylation probe for CTSD gene selected by our model is not present in TCGA cohort

Next, we built a generalized linear Cox model for the accurate prediction of survival using the methylation profile. Using our cohort as a training set (*n* = 60), we built a survival model based on eight genes (Table 1, Fig. 3a, and Supplementary Figure 3). The coefficients of our model contribute to the risk score where negative and positive coefficients in the Cox regression model signify factors that contribute to favorable and unfavorable survival, respectively. In other words, high methylation level of genes with a negative coefficient reduces risk factors. For example, *LTBR*, a gene with a negative coefficient in our model, is associated with tumor necrosis and its activation is linked to carcinogenesis [23]. Thus, the promoter hypermethylation-derived transcription silencing of this gene would be beneficial for PFS. In contrast, a high methylation level of genes with a positive coefficient increases the risk score. For example, *CD3E* is involved in T cell development and activation [24]. The hypermethylation of this gene will result in the negative regulation of T cell-mediated cytotoxicity, which can be expected to be unfavorable for survival.

**Table 1:** Genes used in methylation-based prognostic prediction model

Probe	Coefficient	Gene symbol	Gene function	Cancer relatedness
cg22029157	− 1.52	IRF6	Interferon regulatory factor associated with cytokine signaling	Tumor suppressor activity
cg12007048	− 1.096	CTSD	Proteolytic activation of hormones and growth factors (i.e., EGFR)	Linked with poor prognosis in NSCLC
cg07935119	− 1.04	GRN	Granulin coding gene; growth factor involved in inflammation and cell proliferation	Regulate tumorigenesis; immune evasion, proliferation
cg23079808	− 0.989	LTBR	Tumor necrosis factor receptor; signaling immune response and programmed cell death	Activation linked to carcinogenesis
cg04450862	− 0.907	TRIM36	Mediate ubiquitination and proteasomal degradation of target protein	Known to be upregulated in cancer
cg19918549	0.347	EVL	Actin-associated proteins involved in axon guidance	Upregulated in breast cancer
cg24612198	0.385	CD3E	Part of CD3 complex that facilitate T cell development	Down regulation of this gene results in T cell apoptosis
cg17771150	0.969	LCP1	Actin-binding protein that are involved in T cell activation	Upregulated in cancer

We validated our model using two independent cohorts including TCGA NSCLC data (Fig. 3b–d). Although the TCGA samples in general are not appropriate to evaluate immunotherapy markers, some tumors under strong immune pressure can serve as surrogates for samples treated with checkpoint blockade. Therefore, we selected samples with high immune cell infiltration using tumor-infiltrating lymphocyte (TIL) fraction and tested whether our methylation markers are associated with the survival of these samples [25]. As a result, our model demonstrates its predictive power to accurately distinguish survival outcomes in immunotherapy and TCGA high-TIL cohorts (Fig. 3a–c) but not in TCGA low-TIL cohort (Fig. 3d). For patients with a low risk score and better survival, genes with a negative coefficient had a high level of methylation and those with positive coefficient have low level of methylation (heatmaps in Fig. 3). This trend is opposite for patients with high risk scores. We also performed receiver operating characteristic (ROC) curve analysis of the risk score for predicting progression-free survival (Supplementary Figure 4). The area under curve (AUC) values at 6 and 12 months are consistently higher in immunotherapy (SMC and IDIBELL) and TCGA high-TIL cohorts than TCGA low-TIL cohort. The AUC values in immunotherapy and TCGA high-TIL cohorts ranged from 0.60 to 0.87, while the values in TCGA low-TIL cohort were close to 0.5, indicating no predictive effect of our risk score in this low-immune pressure cohort (i.e., random performance). Collectively, our results reinforce the role of the risk score as a potential biomarker of response to immunotherapy. Interestingly, each gene used in our model can also distinguish survival outcomes in immunotherapy cohorts (Supplementary Figure 5). Consistent with our regression model, genes with a negative coefficient show an inverse relationship between methylation and survival whereas those with a positive coefficient show a direct association. Thus, differential patterns of these genes contribute to the accurate classification of patients’ survival outcome prediction.

### Mutually exclusive hypermethylated genes are associated with patient survival

Previous studies have revealed mutually exclusive patterns of genomic alterations across cancer patients, including mutations in driver genes [26]. Genes exhibiting mutually exclusive alternation pattern are generally involved in a common biological pathway due to their functional redundancy [27, 28]. Knowledge about these patterns can provide important insight into novel cancerous network and potential therapeutic targets [29]. To identify such mutually exclusive patterns associated with response to ICB therapy at the methylation level, we first integrated two different cohorts receiving immunotherapy (i.e., the IDIBELL cohort and SMC cohort; *n* = 141) and identified genes for which promoter hypermethylation is specifically observed in nonresponders (*n* = 101; see “Methods” section). We then searched for protein interaction networks whose member genes show mutually exclusive alteration patterns. We identified 474 protein interaction networks in which two or three hypermethylated genes showed significantly mutually exclusive patterns across nonresponders. Importantly, we further conducted survival analysis to assess the ultimate effect of exclusivity and identified 30 protein networks associated with progression-free survival (Supplementary Table 5).

**Fig. 4:**
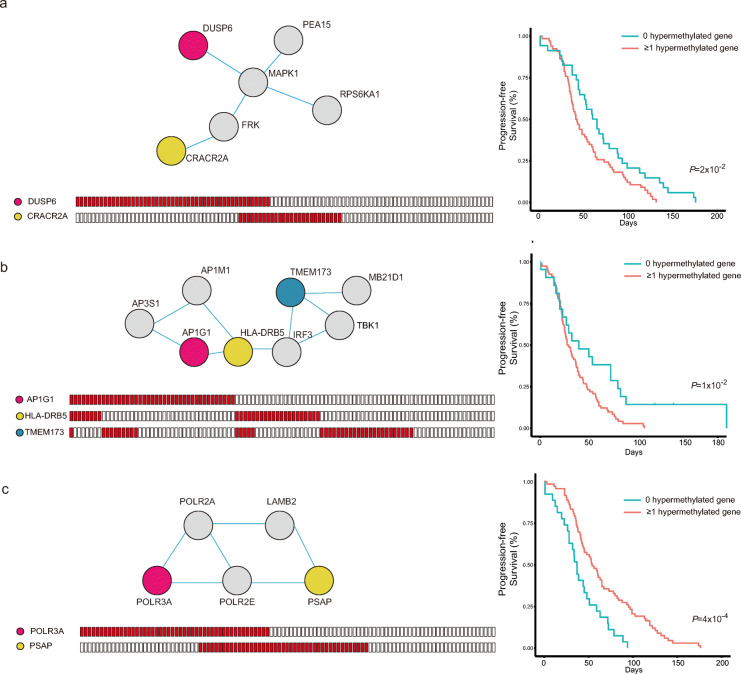
Mutually exclusive alterations associated with nonresponders’ survival. **a**–**c** Representative protein interaction networks showing mutually exclusive hypermethylation patterns. The nodes and edges in the networks represent proteins and interactions, respectively (top right). The colored nodes correspond to genes showing a mutually exclusive promoter hypermethylation pattern. Genes whose promoter is hypermethylated in nonresponders are marked in red (bottom right). Kaplan-Meier survival analyses of the patients are displayed on the right. Patients showing hypermethylation in either or both colored nodes (red) were compared with those without hypermethylation (blue). *P* values were calculated using the log-rank test

Representative networks associated with patient survival are shown in Fig. 4. As shown in Fig. 4a, *DUSP6* and *CRACR2A* showed mutually exclusive patterns (*P* = 3.3 × 10^−6^). In other words, there is a significantly low portion of samples that harbor promoter hypermethylation in both genes. Furthermore, we performed survival analysis by comparing patients with promoter hypermethylation in either or both genes with those without hypermethylation, resulting in an unfavorable prognosis of patients with hypermethylated genes. *DUSP6* is a tumor suppressor gene known to negatively regulate MAPK pathways [30]. Furthermore, recent reports highlight its role in differentiation and apoptosis in cancer. *CRACR2A* is part of the calcium channel in T cells and is pivotal in T cell cytotoxic activity [31]. This result highlights that the disruption of the network caused by the silencing of either gene confers unfavorable prognosis in NSCLC patients.

This pattern can also be found in the network with three mutually exclusive genes (Fig. 4b; AP1G1-TMEM173: *P* = 1.06 × 10^−5^, AP1G1-HLA-DRB5: *P* = 3.4 × 10^−5^, TMEM173-HLA-DRB5: *P* = 1.98 × 10^−5^). *TMEM173* is known to be associated with the apoptotic signaling pathway related to MHC type II, in which *HLA-DRB5* is a subunit [32]. Liu et al. reported that the transcriptional silencing of *AP1G1* can activate the PIK3/AKT pathway, which induces tumor proliferation and invasion [33]. Thus, the promoter hypermethylation of any of the three genes can lead to reduced survival by disrupting the network.

In contrast, the representative network in Fig. 4c shows the opposite survival trend; samples with hypermethylation in either gene have a longer PFS. This finding can also be explained by the gene function of the two mutually exclusive genes, *POLR3A* and *PSAP* (*P* = 1.3 × 10^−5^). *POLR3A* is a RNA polymerase III subunit and is known as an unfavorable marker in cancer [34]. *PSAP* produces prosaposin, whose amplification is reported to cause carcinogenesis or tumor progression [35]. Thus, the disruption of this network by transcriptional silencing can be expected to yield better survival (*P* = 4 × 10^−4^).

## Discussion

DNA methylation aberrations are closely related to cancer since they regulate spatiotemporal gene expression in cell type specific manner. In particular, the downregulation of tumor suppressors by promoter hypermethylation is a widely accepted mechanism in cancer progression [36, 37]. Due to its distinct pattern in cancer, DNA methylation has been used as a diagnostic and predictive biomarker. For example, methylation profiles obtained from blood liquid biopsy have the potential to detect early stage colorectal cancer (CRC) with high efficacy [38]. In addition, a five-CpG-based classifier improved risk stratification for patients with clear cell renal cell carcinomas (ccRCCs) [39]. In this study, we showed that DNA methylation alterations are also a promising biomarker for predicting the clinical response of ICB therapy. We first characterized methylation differences between responders and nonresponders on a genome-wide scale and demonstrated that gene promoter hypermethylation significantly correlates with the mRNA expression silencing of immune-related pathways in nonresponders. We built and validated an eight-gene based lasso regression prediction model to accurately classify patient survival outcomes. The established model can be used to stratify patients who are likely to benefit from ICB therapy. Furthermore, through mutually exclusive hypermethylation pattern analysis, we found protein interaction networks in which exclusive disruption leads to different survival outcomes. The wide range of genes identified in this study has broaden knowledge about potential immunotherapy resistance mechanism. More investigation is warranted in order to understand the mechanisms underlying the associations between genes.

A growing body of evidence suggests that DNA methyltransferase inhibitors (DMTis), such as 5-azacytidine (AZA) and 5-aza-2’- deoxycytidine (DAC), have potential to improve the therapeutic efficacy of ICB [40, 41]. DMTi treatment can promote anti-tumor immunity by upregulating immune-related genes [42] or endogenous retrovirus (ERV) [43]. Our results warrant future research on the effectiveness of the combination of ICB therapy with DNMi because a significant proportion of immunomodulatory pathways might be potentially downregulated in nonresponders by promoter hypermethylation. The identified methylome-based markers could be further used to guide the selection of patients for this combined therapy in clinical trials.

## Conclusions

Our study demonstrates that methylation patterns can provide insight into molecular determinants underlying the clinical benefit of ICB therapy.

## Methods

### Data collection

Advanced NSCLC patients who received anti-PD-1/PD-L1 therapy (*n* = 60) were registered for this study at Samsung Medical Center. This study was approved by Samsung Medical Center’s institutional review board (2018-03-130 and 2013-10-112). The clinical benefit was determined according to RECIST v1.1 [44]. If the patient experienced a partial response (PR) or stable disease (SD) of more than 6 months, he or she was categorized as responder receiving durable clinical benefit (DCB). Conversely, if a patient experienced progressive disease (PD) or SD of less than 6 months, he or she was categorized as non-responder with non-durable benefit (NDB). Of the 60 patients, 14 were responders (DCB), 44 were nonresponders (NDB), and 2 were not determined (NA). The processing of DNA methylation (850K Infinium Methylation EPIC Array), exome- and RNA-seq (*n* = 27) data was illustrated in our previous study [45]. Neo-antigen load was calculated using DeepNeo [46]. Aneuploidy level was retrieved from our previous research [45].

Methylation profiles (850K Infinium Methylation EPIC Array) and matching clinical information of 81 patients with stage IV NSCLC treated with anti-PD-1 medication were retrieved from the research performed by the Esteller et al. group (IDIBELL cohort) [47]. The methylation (Illumina Infinium HumanMethylation 450K) and the clinical profiles of 479 lung adenocarcinoma (LUAD) and lung squamous cell carcinoma (LUSC) patients from The Cancer Genome Atlas (TCGA) were obtained from the publication page (https://gdc.cancer.gov/about-data/publications/pancanatlas).

### Identification of differentially methylated/expressed pathways between nonresponders and responders

We first assigned the most differently methylated promoter probes between responders and nonresponders into a corresponding gene. Promoter probes were defined as those that reside in either the transcription start site (TSS), 1^st^ exon, or 5’UTR region. Ranked genes by the t-static (methylation level difference between nonresponders and responders) were used for input into the preranked module of the GSEA software with Reactome pathways (provided at https://reactome.org/) belonging to the Immune System category (*n* = 181). We also ranked genes by mRNA expression difference between nonresponders and responders and then used the same procedure to identify differentially expressed pathways. DMRs were calculated as regions with differential methylation patterns with three minimum probes and 500 maximum gap using the ChAMP package [48].

### Building a prediction model of clinical benefit

We fitted the Cox model based on elastic-net to predict clinical benefit using the glmnet R package [49]. For efficient computation, we narrowed down the methylation probes with two criteria. First, we selected probes related to known immune genes curated by Reactome version 69. Second, probes showing significant differences (*P* < 5 × 10^−4^ by *t* test) between responders and nonresponders were selected. After filtering, 18 probes were given as input to the lasso-regularized Cox proportional hazards model, resulting in eight probes in different genes in our final model (Table 1). For PSW-1711, whose survival information was not available, we treated this case with a modest standard as having no progression for 1 day.

The model’s prediction power was validated on two independent data sets: IDIBELL and TCGA NSCLC cohorts. For 410 TCGA samples whose tumor-infiltrating lymphocyte (TIL) fraction is available, we divided them into high- (*n* = 151) and low-immune pressure groups (*n* = 259) according to mean value of TIL fraction [25]. The patients in each cohort were divided into low- and high-risk groups based on the risk index produced by our model (i.e., mean score). The risk index in the validation cohorts was calculated using the predict function of glmnet. A heatmap of eight genes (Fig. 4) was drawn using the heatmap.2 function in the gplots package [50].

### Mutually exclusive hypermethylation pattern analysis

A total of 141 samples from our cohort and IDIBELL cohort were combined using ComBat, a tool that adjusts batch effects when combining two different datasets [51]. In the combined dataset, 40 patients were responder, and 101 were nonresponders. We first identified hypermethylated genes in nonresponders for which the methylation level was greater than two standard deviations from the mean in responders. The data were further reduced by several factors. First, hypermethylated genes with more than 10 occurrences (approximately 10% of nonresponders) were selected to see general patterns among nonresponders. Second, immune related genes in the Reactome were selected to focus on immunologic patterns. Using WExT, a tool that searches for mutually exclusive patterns, we found 472 mutually exclusive gene sets comprised of two or three genes [52]. Then, nonresponders were divided into two groups: one group is comprised of samples with no hypermethylation in either gene and the other with one or more hypermethylated genes. Using the survival R package, we performed survival analysis on each set and discovered 30 pairs with differential survival between samples with and without hypermethylation [53]. Networks of mutually exclusive genes were created using STRING and GeneMANIA, tools that predict functional interaction networks using multiple databases [54, 55].

## Supplementary information

**Additional file 1: Figure S1.** Survival plots of previously known biomarkers. (a) tumor mutation burden (b) neo-antigen load (c) aneuploidy level (d) PD-L1 expression. Mutation burden, neo-antigen load, and aneuploidy level were calculated with exome-sequencing data (n = 60) and PD-L1 expression was obtained from RNA-sequencing data (n = 28).

**Additional file 2: Figure S2.** Summary statistics of differentially hyper- (a), and hypomethylated (b) probes, and hypermethylated regions (c) in nonresponders according to their location. Differentially hypomethylated regions are now shown due to the lack of them.

**Additional file 3: Figure S3**. Tuning parameter log (lambda) selection during ten-fold cross validation (a) and L1-norm value (b) used for the regression model.

**Additional file 4: Figure S4.** ROC curve analysis of the risk score for predicting progression-free survival greater than six months (a) and one year (b).

**Additional file 5: Figure S5.** X-tile plots of the methylation patterns of the eight genes used in the regression model for a total of 141 samples across immunotherapy (SMC and IDIBELL) [56]. Each plot represents an association between individual gene methylation level and survival. The cut point, which separates high- and low-risk groups, represents the highest *χ*^2^-value obtained from the Kaplan-Meier survival analysis and log-rank test. Green and red indicate direct and inverse relationships, respectively, between methylation and survival.

**Additional file 6.** Supplementary tables

## Data Availability

Our cohort dataset (methylation chip and RNA-seq data) is available at Gene Expression Omnibus under accession number GSE119144 and GSE135222. The raw data for the exome sequencing of our cohort is available at European Genome-phenome Archive under accession number EGAS00001003731.

## Abbreviations

ICB: Immune checkpoint blockade
NSCLC: Non-small-cell lung cancer
DMR: Differentially methylated regions
TMB: Tumor mutational burden
PFS: Progression-free survival
DMTis: DNA methyltransferase inhibitors
