# Supplementary figures and images for: Genome-wide methylation patterns predict clinical benefit of immunotherapy in lung cancer
Source: Clin Epigenetics. 2020 Aug 6;12:119. doi: 10.1186/s13148-020-00907-4 (PMC7410160; doi:10.1186/s13148-020-00907-4)

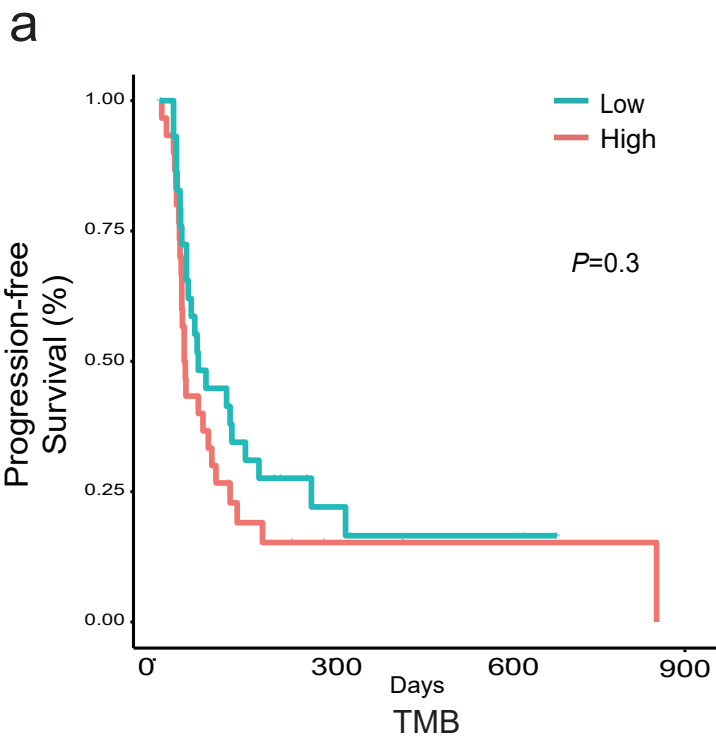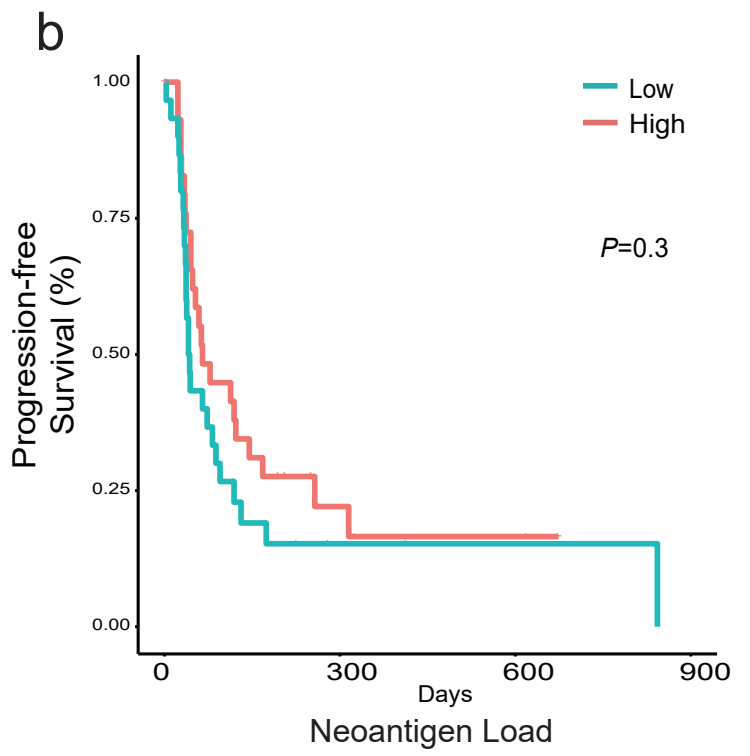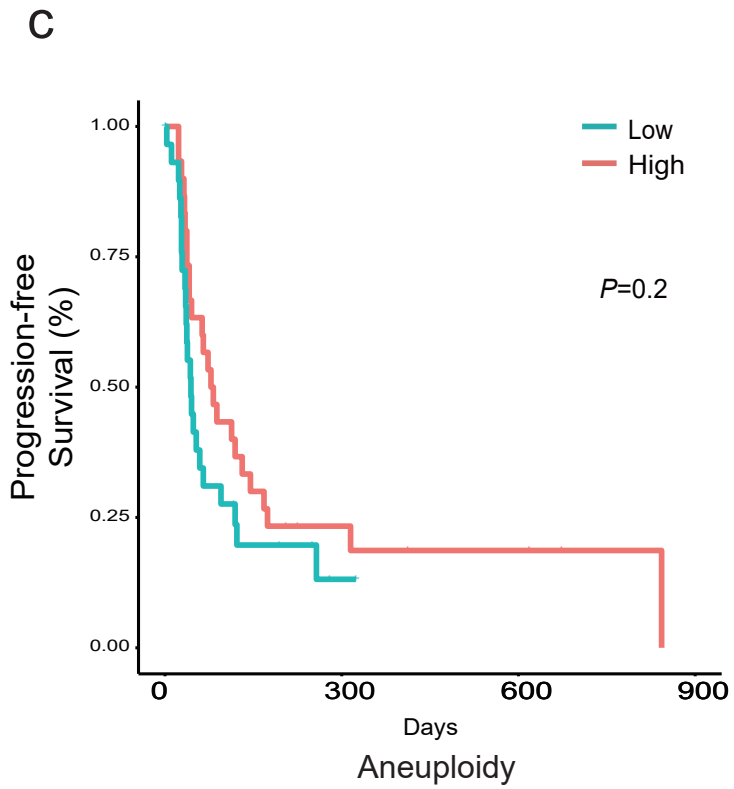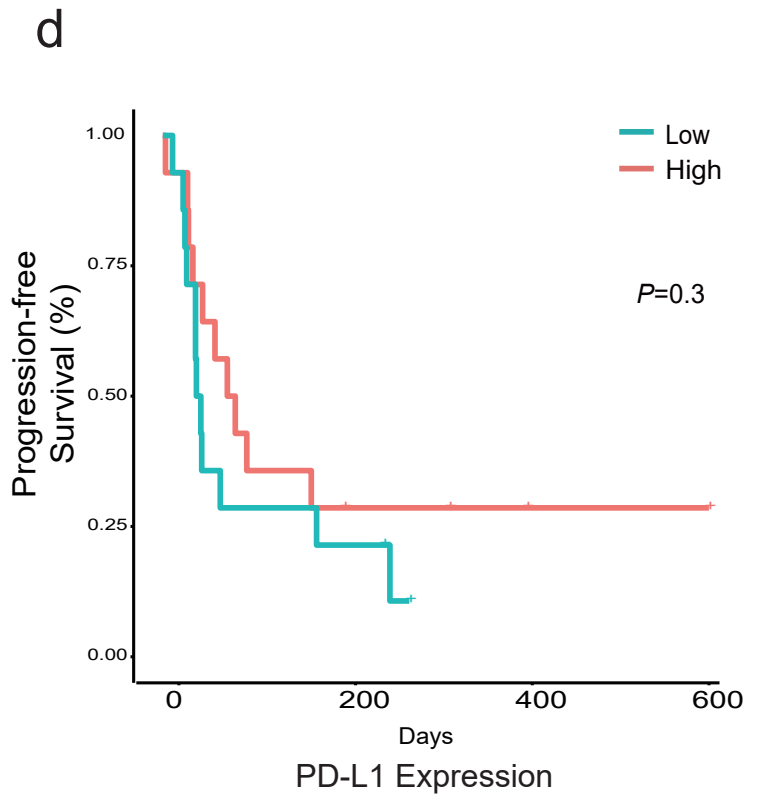

Supplement: Supplementary file 1 — Additional file 1: Figure S1. Survival plots of previously known biomarkers. (a) tumor mutation burden (b) neo-antigen load (c) aneuploidy level (d) PD-L1 expression. Mutation burden, neo-antigen load, and aneuploidy level were calculated with exome-sequencing data (n = 60) and PD-L1 expression was obtained from RNA-sequencing data (n = 28). [file 13148_2020_907_MOESM1_ESM.pdf]

a

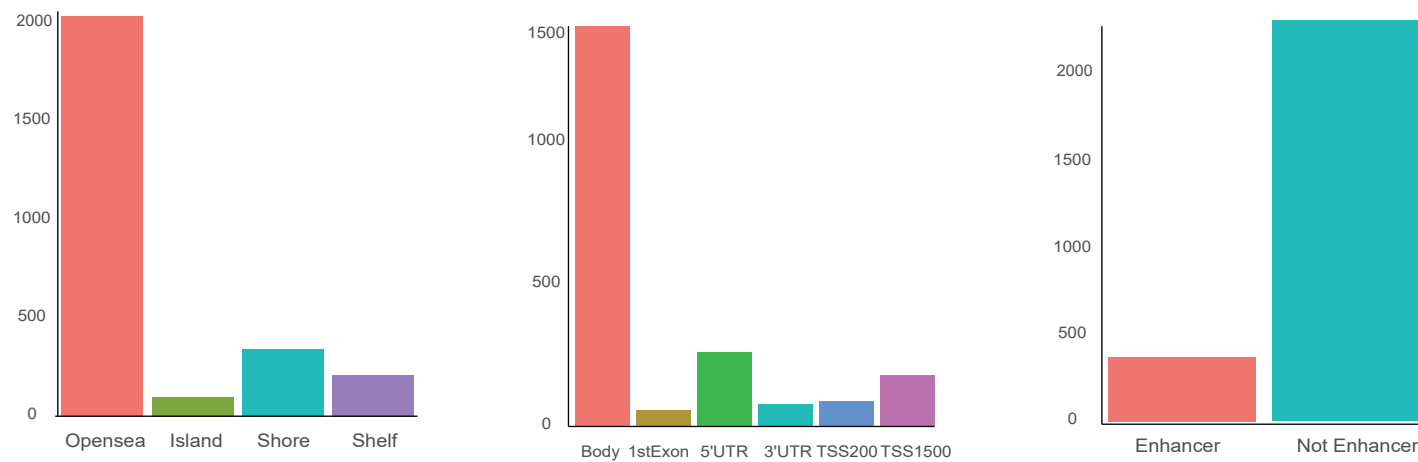

b

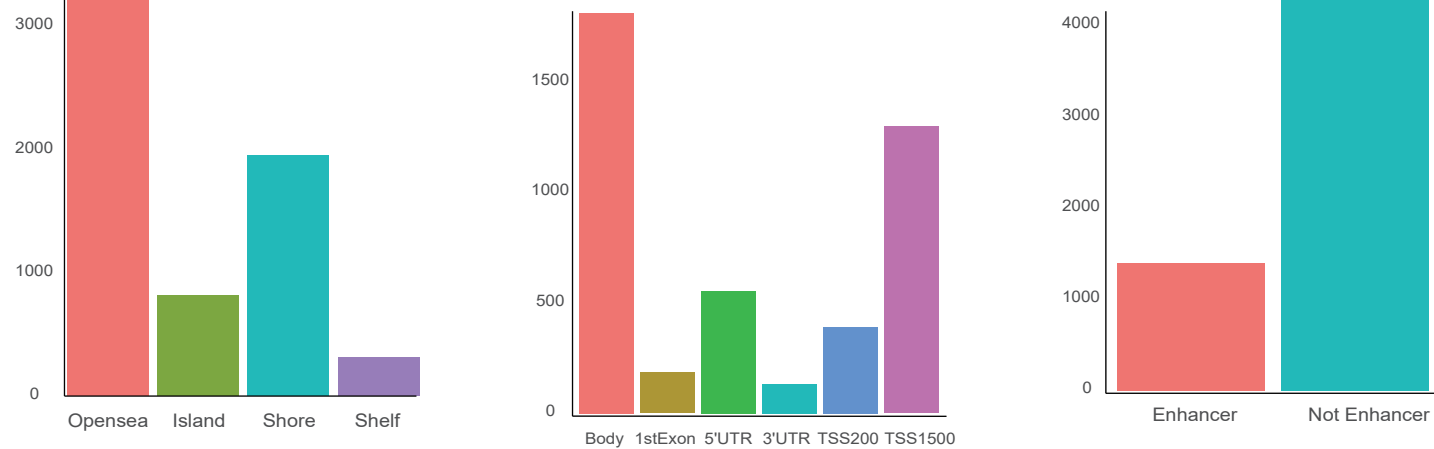

c

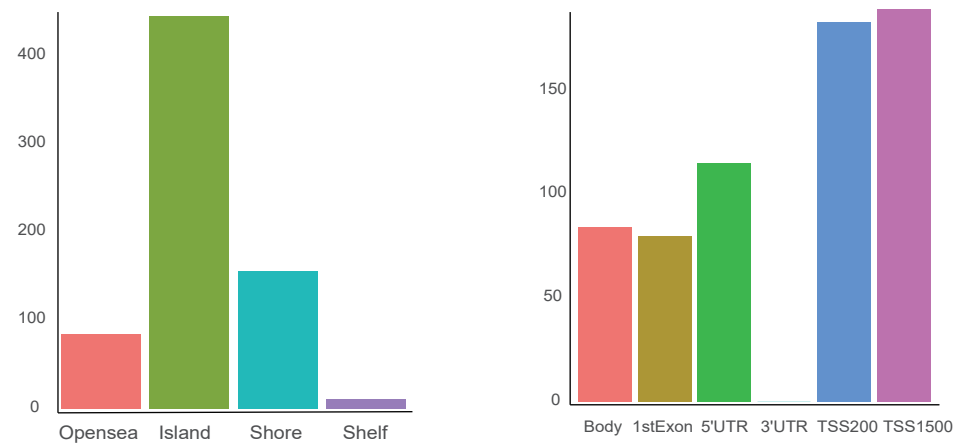

Supplement: Supplementary file 2 — Additional file 2: Figure S2. Summary statistics of differentially hyper- (a), and hypomethylated (b) probes, and hypermethylated regions (c) in nonresponders according to their location. Differentially hypomethylated regions are now shown due to the lack of them. [file 13148_2020_907_MOESM2_ESM.pdf]

a

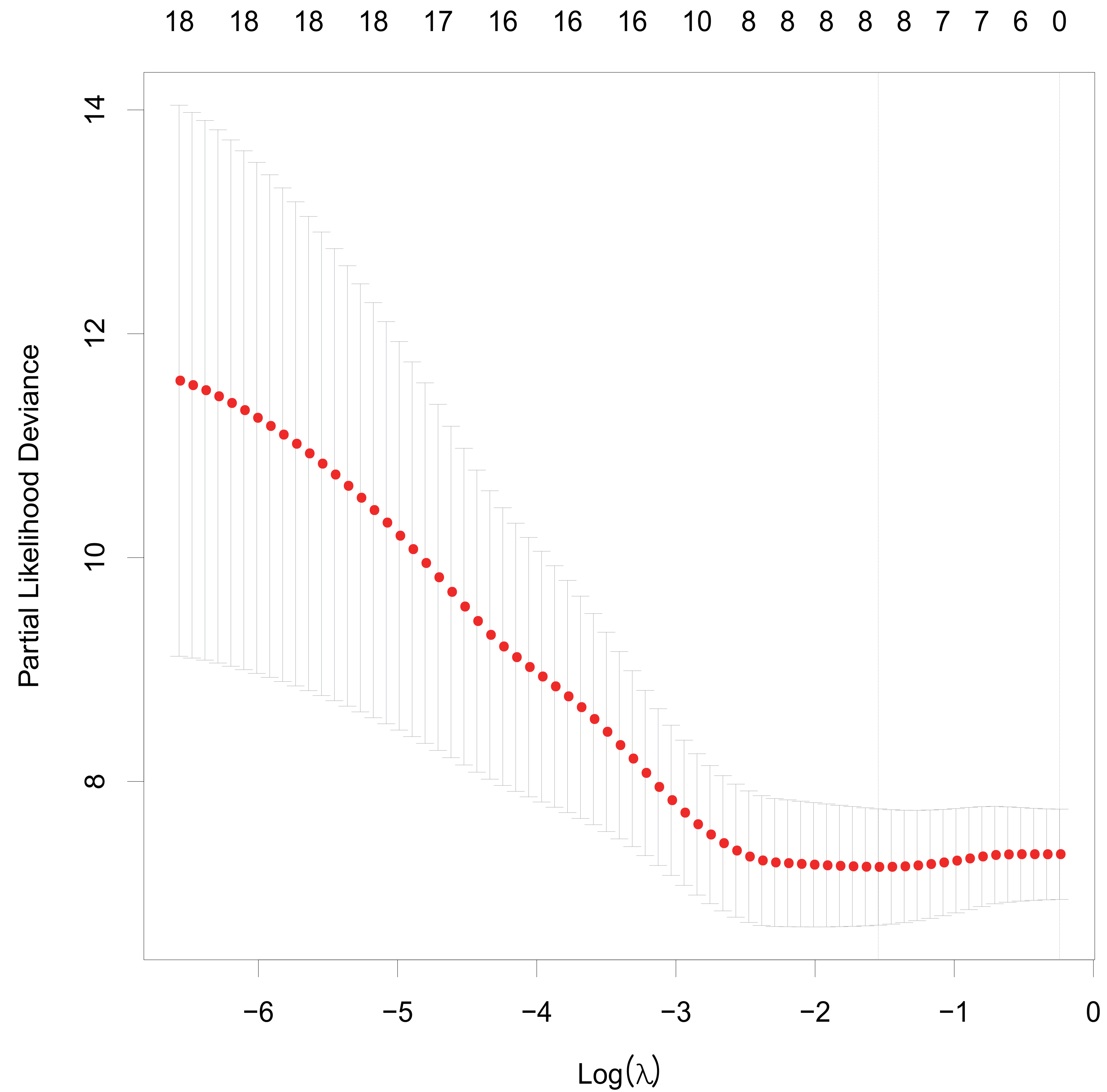

b

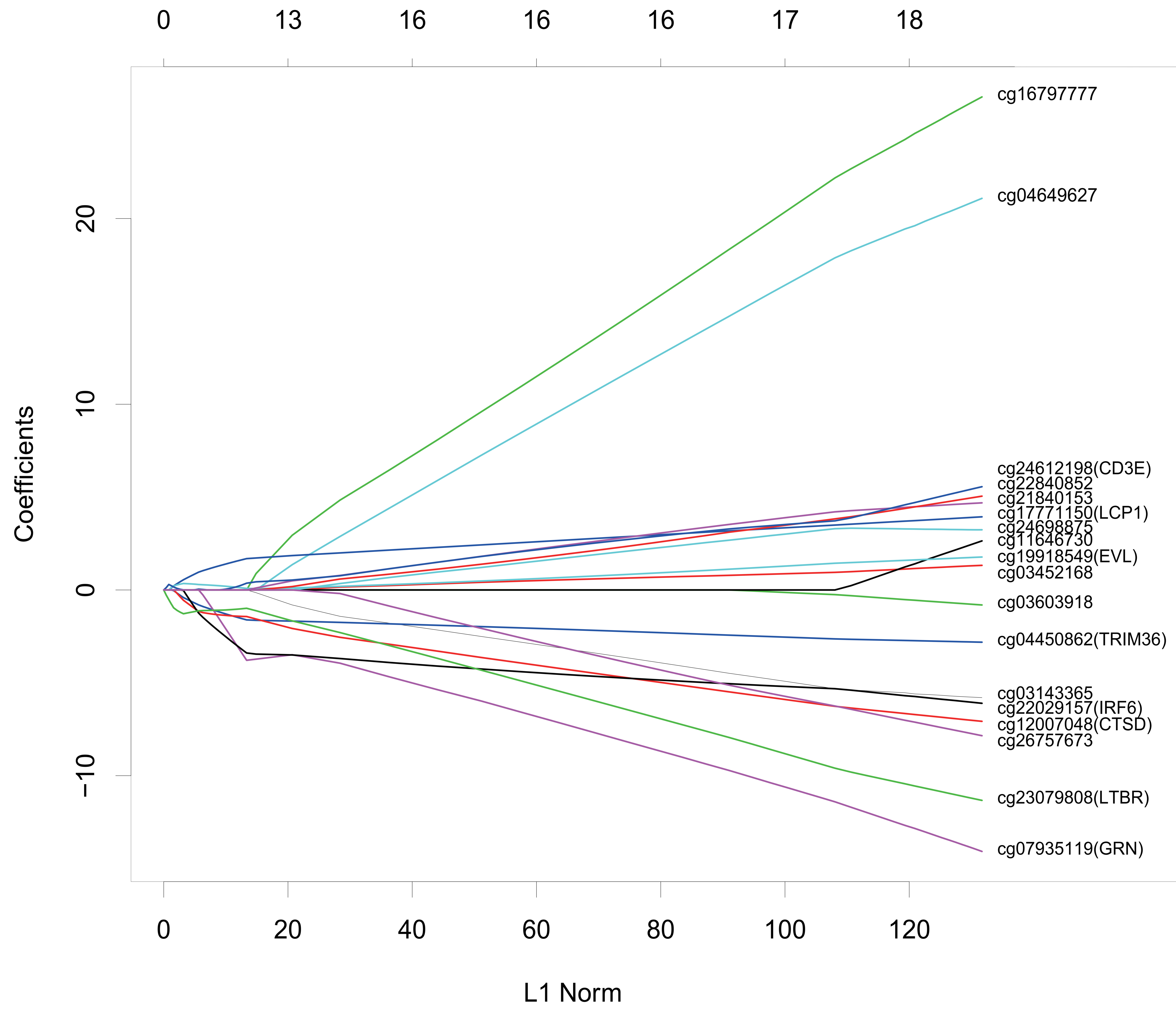

Supplement: Supplementary file 3 — Additional file 3: Figure S3. Tuning parameter log (lambda) selection during ten-fold cross validation (a) and L1-norm value (b) used for the regression model. [file 13148_2020_907_MOESM3_ESM.pdf]

a

ROC 6 months

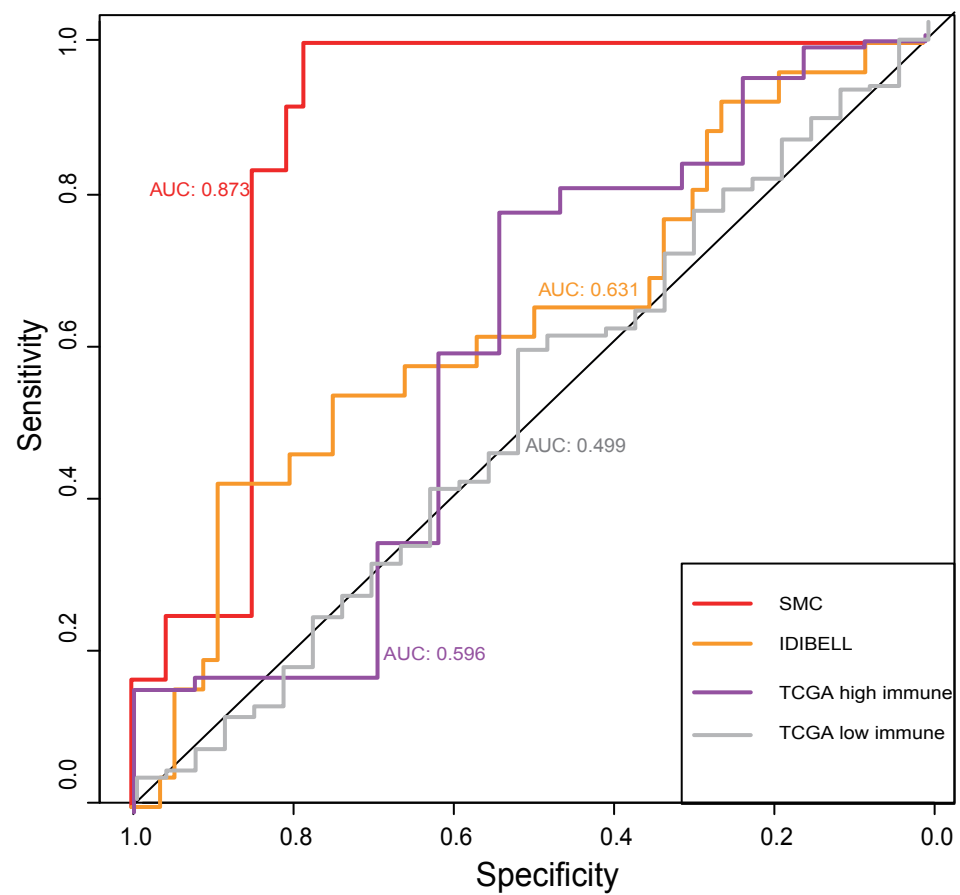

b

ROC 12 months

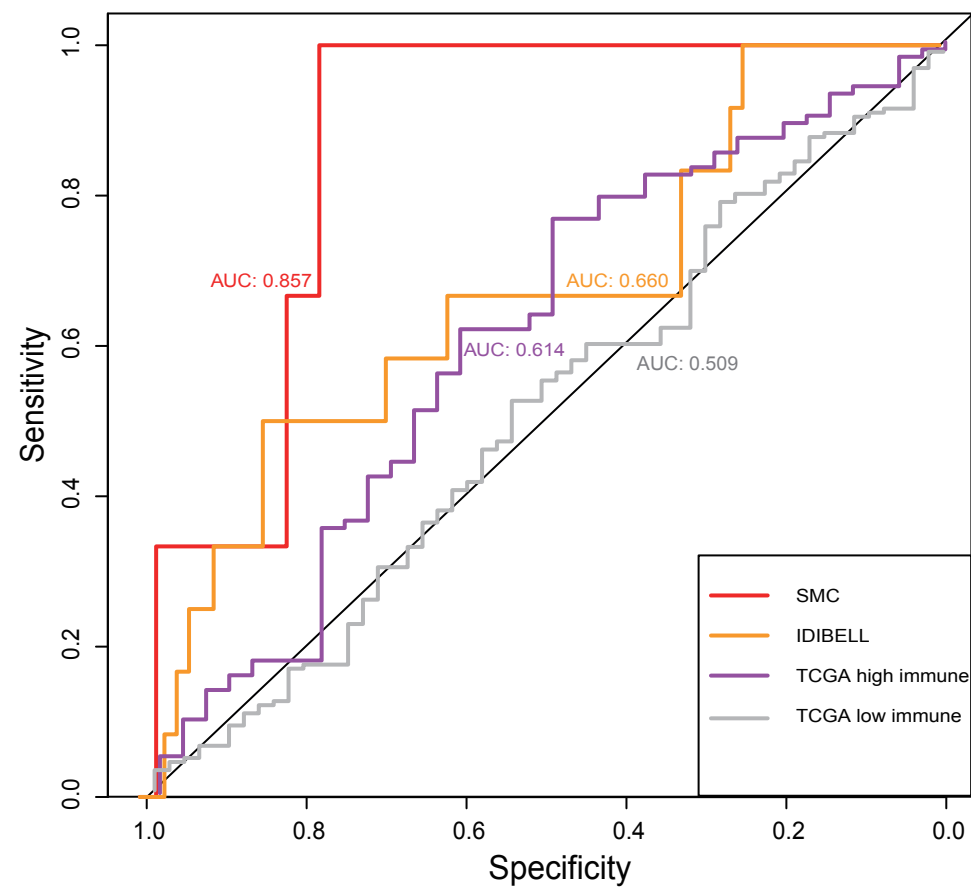

Supplement: Supplementary file 4 — Additional file 4: Figure S4. ROC curve analysis of the risk score for predicting progression-free survival greater than six months (a) and one year (b). [file 13148_2020_907_MOESM4_ESM.pdf]

IRF6

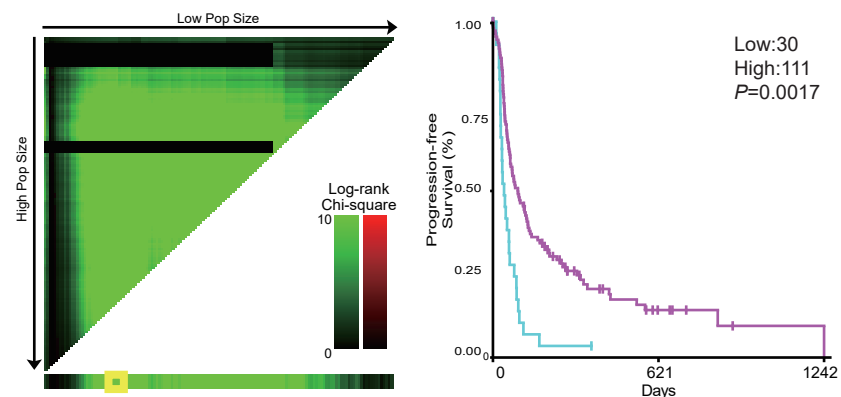

CTSD

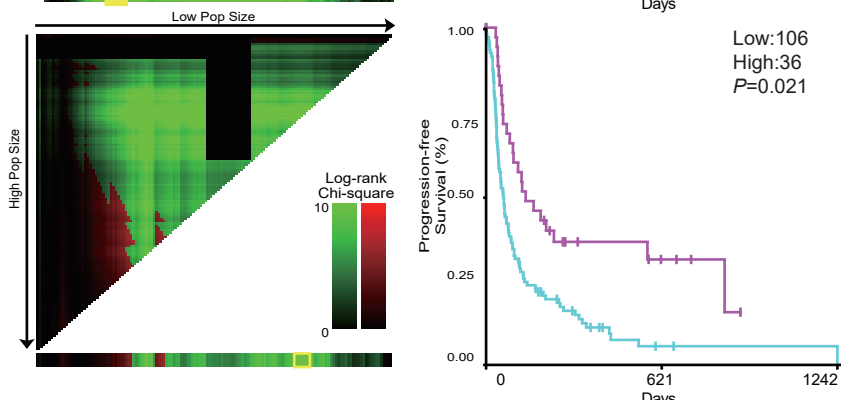

GRN

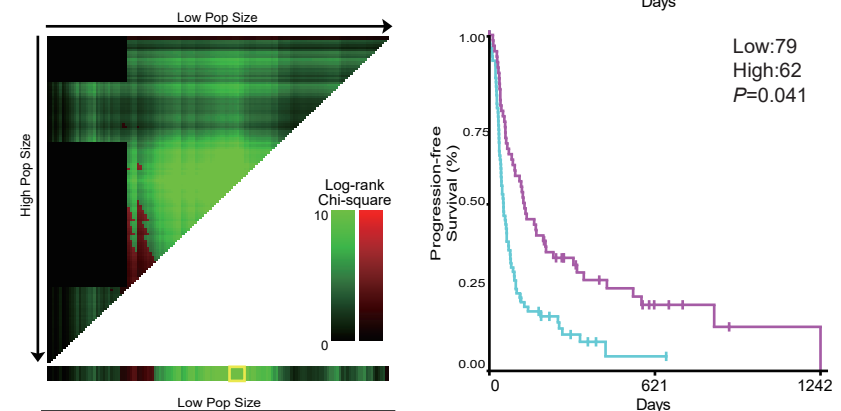

LTBR

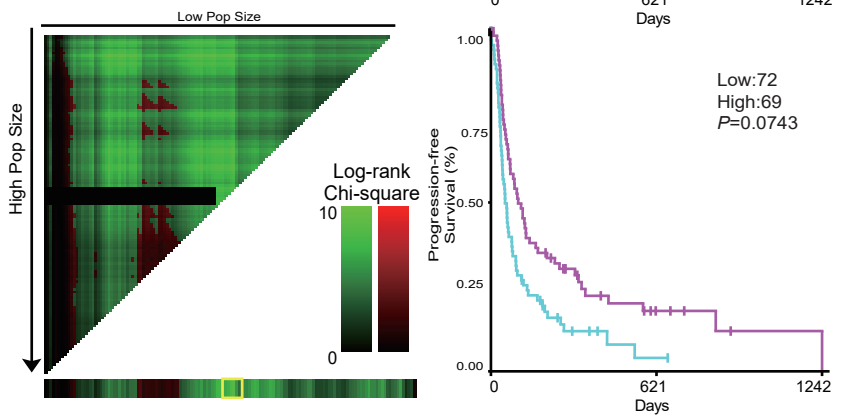

TRIM36

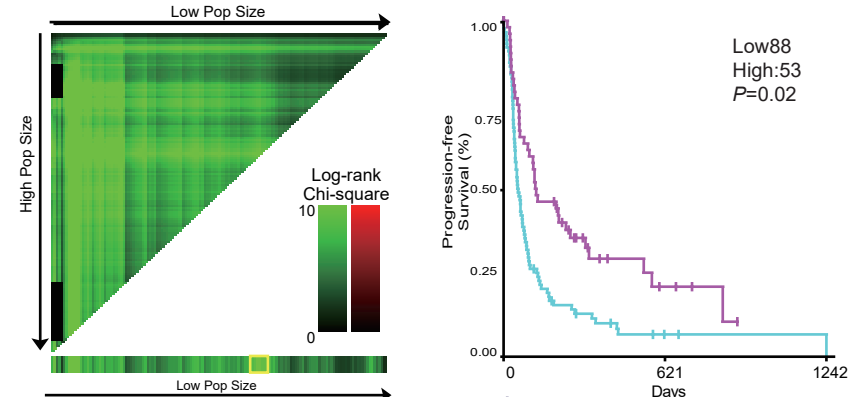

EVL

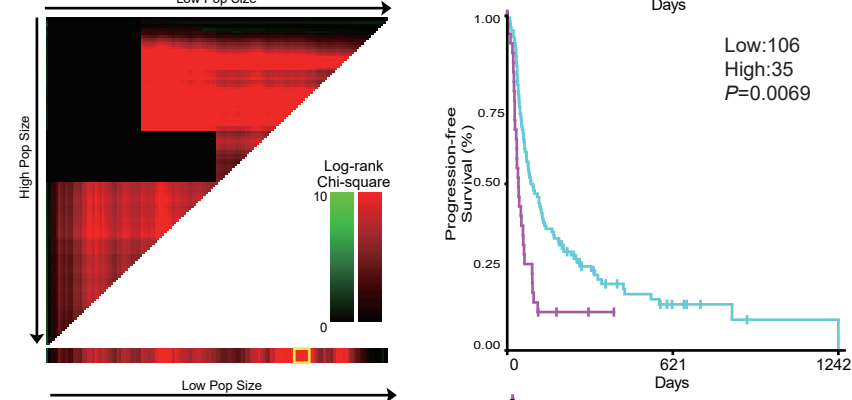

CD3E

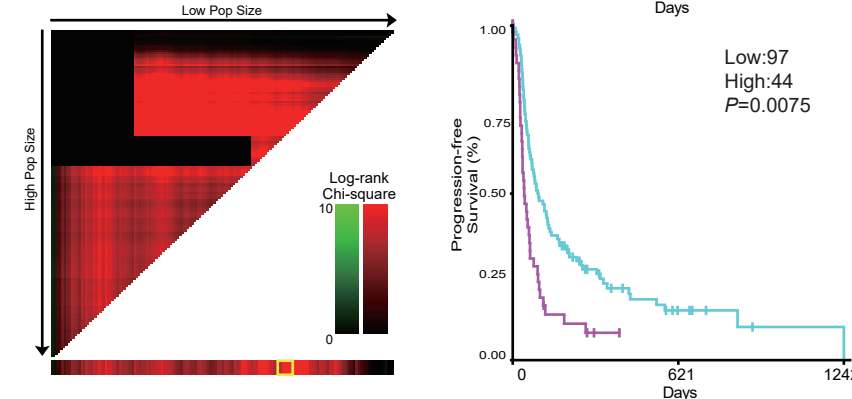

LCP1

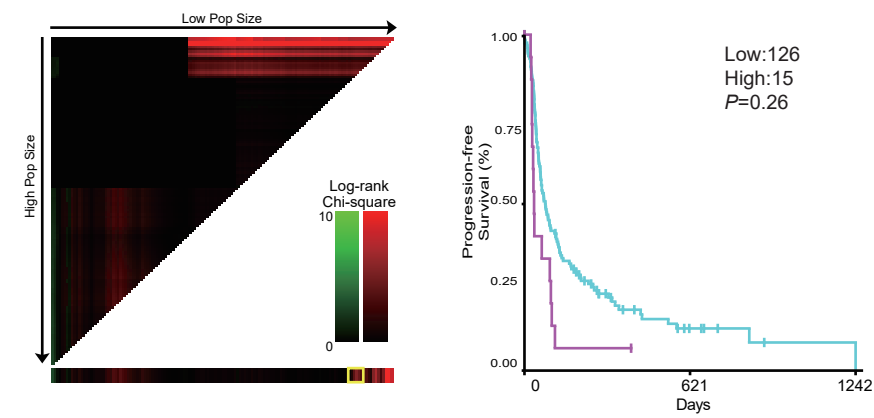

Supplement: Supplementary file 5 — Additional file 5: Figure S5. X-tile plots of the methylation patterns of the eight genes used in the regression model for a total of 141 samples across immunotherapy (SMC and IDIBELL) [56]. Each plot represents an association between individual gene methylation level and survival. The cut point, which separates high- and low-risk groups, represents the highest χ2-value obtained from the Kaplan-Meier survival analysis and log-rank test. Green and red indicate direct and inverse relationships, respectively, between methylation and survival. [file 13148_2020_907_MOESM5_ESM.pdf]
